# Effect of gestational age and postnatal age on the endothelial glycocalyx in neonates

**DOI:** 10.1038/s41598-021-81847-8

**Published:** 2021-02-04

**Authors:** Alexandra Puchwein-Schwepcke, Stefanie Artmann, Lea Rajwich, Orsolya Genzel-Boroviczény, Claudia Nussbaum

**Affiliations:** Division of Neonatology, Dr. von Hauner Children’s Hospital, University Hospital, LMU Munich, Lindwurmstr. 4, 80337 Munich, Germany

**Keywords:** Medical research, Risk factors

## Abstract

Prematurity predisposes to cardiovascular disease; however the underlying mechanisms remain elusive. Disturbance of the endothelial glycocalyx (EG), an important regulator of vessel function, is thought to contribute to vascular pathology. Here, we studied the EG with respect to gestational and postnatal age in preterm and term neonates. The Perfused Boundary Region (PBR), an inverse measure of glycocalyx thickness, was measured postnatally in 85 term and 39 preterm neonates. Preterm neonates were further analyzed in two subgroups i.e., neonates born < 30 weeks gestational age (group A) and neonates born ≥ 30 weeks (group B). In preterm neonates, weekly follow-up measurements were performed if possible. PBR differed significantly between preterm and term neonates with lowest values representing largest EG dimension in extremely premature infants possibly reflecting its importance in fetal vascular development. Linear regression revealed a dependence of PBR on both, gestational age and postnatal age. Furthermore, hematocrit predicted longitudinal PBR changes. PBR measured in group A at a corrected age of > 30 weeks was significantly higher than in group B at birth, pointing towards an alteration of intrinsic maturational effects by extrinsic factors. These changes might contribute to the increased cardiovascular risk associated with extreme prematurity.

## Introduction

Premature neonates and especially those with very low birth weight (< 1500 g) are known to have an increased risk for cardiovascular disease including metabolic syndrome as adults^1^. Adolescents and young adults who were born preterm have higher systolic and diastolic blood pressure and impaired glucose tolerance compared to adults born full-term^2–5^. Already in childhood, increased intima media thickness as a marker for early arteriosclerosis and greater vessel stiffness have been observed in former preterm infants^6–8^.

The mechanisms underlying this increased cardiovascular risk are not well defined. Alterations in microvascular architecture and function associated with prematurity may play a role in the development of hypertension^9,10^. In a previous study, we were able to demonstrate that premature neonates exhibit a distinct microvascular phenotype compared to mature neonates that persists throughout the first month of life and even into adolescence^6,11^. Furthermore, several studies reported a disturbed endothelial function—considered a key factor in the development of vascular disease-in low birth weight neonates^12–15^.

In the last years, the endothelial glycocalyx (EG) has been recognized as an essential determinant of vascular integrity and health. This delicate structure composed of proteoglycans, glycoproteins, glycosaminoglycans and associated plasma proteins covers the endothelial cells of the whole vasculature. It is involved in important vascular functions such as regulation of vessel tone, permeability, cell-endothelial interaction and hemostasis as reviewed by Cosgun et al.^16^. Destruction and/or shedding of the EG have been witnessed in various acute and chronic disease states in children and adults including sepsis, hyperlipidemia, diabetes mellitus, and cardiopulmonary bypass, all of which are characterized by vascular dysfunction^17–22^. However, so far the EG has not been measured in newborns. Therefore, it was the aim of the present study to (1) compare the EG of mature and premature neonates at various gestational ages after birth, (2) to assess a possible association between the EG and gestational age at birth, (3) to longitudinally evaluate postnatal changes in the EG in preterm infants, (4) identify potential influencing factors by correlation of EG measurements with clinical and laboratory data.

## Methods

### Study design and patient recruitment

The study was conducted as a single-center prospective observational study at the Perinatal Center Innenstadt of the Ludwig-Maximilians-University, Munich, Germany. Term neonates (≥ 37 weeks of gestation) were recruited after birth on the maternity ward periodically between Dec 2013 and Jan 2016. Premature infants (gestational age < 37 completed weeks of gestation) were recruited between Dec 2013 and Dec 2014 upon admission to the neonatal intensive care unit. For subgroup analyses, preterm neonates were divided into infants born < 30 + 0 weeks of gestation (Group A) and infants born ≥ 30 + 0 – 36 + 6 weeks of gestation (Group B). Exclusion criteria were significant congenital malformations including complex heart defects, suspected syndromal disease and dark skin pigmentation (due to technical limitations in visualizing the microcirculation with SDF imaging through pigmentation of melanocytes).

### Microvascular recordings and assessment of the endothelial glycocalyx

**Figure 1:**
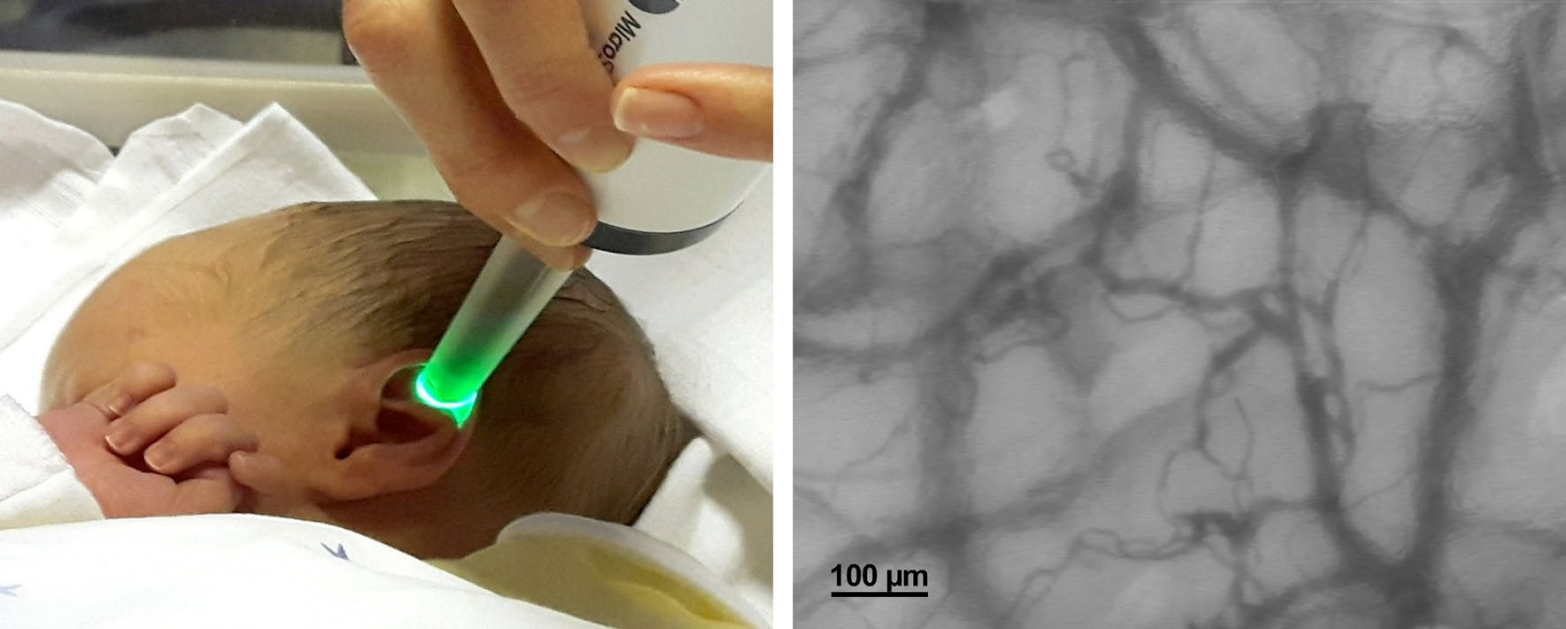
Visualization of the cutaneous microcirculation in a mature neonate using SDF-imaging technology.

Intravital recordings of the skin microcirculation were obtained at the fossa triangularis of the ear using Sidestream Dark Field (SDF) imaging technology as described before^18^. The SDF technique allows non-invasive visualization of the microvasculature at the bedside (Fig. 1).The device consists of a handheld video microscope (MicroScan 5×/0.2; MicroVision Medical Amsterdam, The Netherlands) with ring LEDs emitting pulsed green light at a wavelength of 530 nm (= isobestic point in the absorption spectrum of hemoglobin). The light is absorbed by erythrocytes, but scattered by the tissue and collected via a central light guide, leading to an on-screen image of dark vessels against a light background with a resolution of ~ 1 µm/pixel^23^.

A specialized software (GlycoCheck, Microvascular Health Solutions Inc., Salt Lake City, UT, USA) was used to calculate the *Perfused Boundary Region* (PBR; µm) as a surrogate for the EG dimension/integrity^24,25^. The software automatically detects vessels based on their contrast and divides them into 10 µm vascular segments. These vascular segments are further divided by 21 perpendicular line markers in 0.5 µm steps. After a quality check, the program measures the width of the red blood cell column (RBCW) on the basis of the light intensity profile along the line markers within at least 3000 valid vessel segments over the duration of 40 frames (i.e., 840 light intensity profiles per vascular segment). Based on the distribution of the RBCW, the median RBCW is calculated whereas the maximum RBCW is derived by linear regression. The PBR, representing the most luminal part of the EG penetrable to erythrocytes, is defined as ½ × (maximum − median RBCW). A large EG limits lateral movements of red blood cells, leading to a small PBR. When the glycocalyx is shed or disturbed, red blood cells can move closer to the vessel wall, represented by an increase in PBR. For each measurement session, the program calculates the mean PBR value for vessels of 5–25 µm diameter.

In addition, the following microcirculatory parameters are provided:

- *Red blood cell filling* (RBC filling; %): percentage of vascular segments filled with erythrocytes over all 40 frames
- *Total vessel density* (TVD; µm/mm^2^): length of all vessel segments identified per field of view.
- *Valid vessel density* (VVD; µm/mm^2^): length of vessel segments with a RBC filling > 50% in the first frame of the video sequence

Automatic recordings only started when preset quality criteria regarding focus, movement and illumination were fulfilled, thus guaranteeing sufficient and constant image quality for further calculations. For every child, 3 (max. 5) separate recordings were performed at each time point, each taking around 1–3 min. Measurements were conducted by two investigators (LR and SA). In nine mature neonates, measurements were obtained by both investigators in parallel to check for interrater reliability as well as three times successively by one single investigator to check for intrarater reliability.

### Measurement schedule

In general, we aimed to schedule microcirculatory measurements when routine blood examinations were performed in order to obtain concomitant laboratory values. Therefore, mature neonates were measured between 36 and 72 h of life together with the newborn screening for inborn errors of metabolism. The first measurement in premature neonates was also obtained at the time of the newborn screening if the general condition of the infant was considered to be clinically stable. A second measurement was done between postnatal days 5–7. Further measurements were planned at weekly follow-up until the child was either at term, transferred to another unit or discharged from the hospital. Despite efforts to combine the follow-up measurements with routine blood sampling, this was not always possible as especially late premature infants did not have weekly routine blood draws, leading to missing laboratory values for some of the measurements.

### Clinical and laboratory data

For every patient, birth-related data such as gestational age, body measures, mode of delivery, APGAR values and cause of prematurity, if applicable, were noted. Furthermore, at every measurement time point the following information was obtained from the patient records: actual weight, postnatal and corrected gestational age and body temperature. Heart- and respiratory rate, oxygen saturation and blood pressure were only routinely monitored in premature neonates.

Laboratory data was obtained by point of care test (POCT) of venous or capillary blood samples including pH, PCO_2_, HCO3^-^, base excess, lactate, hemoglobin, hematocrit, blood glucose and bilirubin. Furthermore, C-reactive protein and Interleukin-6 levels were noted, if available.

### Statistical evaluation

For each time point, the mean of a minimum of 3 measurements per child was used for statistical analysis. Descriptive statistical analyses were performed with GraphPad Prism 8.2 (GraphPad Software, San Diego, CA, USA). Regression models were fitted using Stata software (StataCorp. 2017. Stata Statistical Software: Release 15. College Station, TX: StataCorp LLC., USA; Stata FAQ available at https://www.stata.com/support/faqs/resources/citing-software-documentation-faqs/).

A p value < 0.05 was considered as statistically significant.

Summary statistics were performed to characterize clinical and microcirculatory parameters of term and preterm neonates. The group of preterm neonates was further divided into early and late preterm neonates with 30 + 0 weeks of gestation being the cutoff.

Normal distribution was tested with a D'Agostino–Pearson normality test. To compare frequency distributions a Fisher’s exact test was used. Numeric data of two groups were compared with t test or Mann–Whitney U test, as appropriate. For comparison of more than two groups, an ANOVA or a Kruskal–Wallis test was employed, followed by Holm–Sidak’s multiple comparison test or Dunn’s post hoc test, respectively. To compare changes of a given parameter over time, we used a paired t test or a signed rank test for two time points and a repeated measure ANOVA or a Friedman test for more than two time points, as indicated.

#### Interrater and intrarater reliability

To assess interrater reliability of microcirculatory measurements, a Bland–Altman analysis was chosen. Intrarater reliability was assessed by the coefficient of variation. Pearson and Spearman correlation were used to check for simple correlations of experimental data with clinical and laboratory values.

#### Sequential linear regression model

To answer the question of a possible association between gestational age at birth and PBR, we decided to use linear regression. First, we identified clinical covariates with significant difference between preterm and term neonates and summarized them in a directed acyclic graph illustrating the possible structural relationship between PBR, gestational age at birth, postnatal age and potential confounders (Suppl. Figure S1). Next, we applied a sequential linear regression model to control for potential confounders and then only included statistically significant confounders into the final model.

#### Linear mixed-effects regression model

Our next goal was to assess the development of PBR longitudinally. Therefore, we followed our cohort of preterm neonates over time and measured the glycocalyx once a week. Data was statistically evaluated by means of a linear mixed-effects regression model to take into account the longitudinal, repeated measurements in our cohort. Next, we adjusted for previously identified clinical parameters in a sequential approach.

### Ethical approval

The study was approved by the ethics committee of the medical faculty of the Ludwig-Maximilian-University, Munich, Germany (project 338-13) and registered in the German Clinical Trials Register (DRKS 00022031). Written informed consent was obtained from the parents or legal guardians prior to patient recruitment, and the experiments were carried out in accordance with the Declaration of Helsinki.

## Results

### Patient characteristics

In total, 85 term and 39 premature neonates (Group A: n = 20, Group B: n = 19) were included in the study. Patient characteristics are shown in Table 1 and Suppl. Table S1. The most common reasons for preterm birth were premature labor (n = 23) and/or premature rupture of membranes (n = 21), followed by cervical insufficiency (n = 11). In six cases HELLP- syndrome, pre-eclampsia or pregnancy-related cholestasis was diagnosed.

**Table 1:** Patient characteristics at birth.

	Premature neonates (n = 39)	Mature neonates (n = 85)	P value
Gestational age (weeks)	30.4 (3.5)	40.1 (1.1)	** < 0.0001**
Birth weight (g)	1457 (601)	3512 (448)	** < 0.0001**
Female gender (n)	23 (59%)	43 (51%)	n.s
C-Section (n)	28 (72%)	17 (20%)	** < 0.0001**
APGAR min 5	8.5 (1.8)	9.8 (0.5)	** < 0.001**
APGAR min 10	9.2 (0.9)	10 (0.2)	** < 0.001**
Umbilical artery pH-value	7.34 (0.08)	7.29 (0.09)	**0.0049**

Data are presented as mean (SD) or as number (%) as appropriate. Statistical analysis was performed with t test or Mann–Whitney U test (numerical data) and Fisher’s exact test (categorical data). Statistically significant results are marked in bold.

At birth, eight neonates of the premature cohort showed elevated Interleukin-6 levels, which were followed by a CrP elevation in four cases (10%). Only two preterm infants developed CrP values > 2 mg/dl indicating neonatal sepsis according to NeoKISS criteria^26^. In the group of term neonates, infection was diagnosed in six cases (7%). None of the infants was thought to be septic or critically ill.

### Interrater and intrarater reliability of PBR measurements

As microcirculatory measurements were performed by two investigators, we tested for interrater reliability by Bland–Altman analysis showing a good agreement between both investigators with a mean difference in the PBR values of 0.059 µm and 95% limits of agreement between − 0.25 and 0.36 µm (Suppl. Figure S2). Pearson correlation also demonstrated good correlation (r = 0.84, p = 0.004). Interrater variability between repeated measurements was low with an average coefficient of variation in 3 successive measurements of 7.4% (range 1.5–11.1%).

### Microcirculatory measurements of term neonates

**Figure 2:**
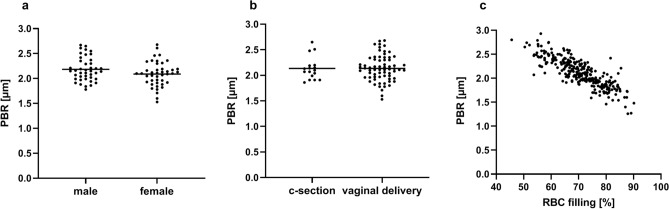
PBR values in mature neonates at 36–72 h of life. (**a**) Gender and (**b**) birth mode did not have a significant influence of PBR values. (**c**) A significant inverse correlation of PBR values and RBC filling was observed (r = − 0.87, p < 0.0001).

Term neonates were measured on average at postnatal day 3.2 and had a mean PBR of 2.14 ± 0.25 µm. Clinical parameters are provided in Table 2 and microcirculatory parameters in Table 3. PBR did not differ between gender (male vs. female) and birth mode (c-section vs. vaginal delivery) (Fig. 2a,b). There was no correlation between PBR and blood glucose (Pearson r = − 0.10, p = 0.37). Hematocrit values in capillary blood gas analyses were significantly higher than in venous samples (64 ± 8% vs. 55 ± 7%; p < 0.001), however, no correlation with PBR (Pearson r = 0.01, p = 0.94) or RBC filling (Pearson r < 0.001, p = 1.0) was observed. Furthermore, we found a highly significant, negative correlation between PBR values and RBC filling (Person r = − 0.87, p < 0.0001) (Fig. 2c).

**Table 2:** Clinical and laboratory data of premature and mature neonates at first measurement.

	Premature neonates (n = 39)	Mature neonates (n = 85)	P value
Day of life at first measurement	5.8 (4.5)	3.2 (0.4)	** < 0.001**
Gestational age at first measurement	30.3 (3.5)	40.1 (1.1)	** < 0.001**
Actual body weight (g)	1417 (570)	3333 (438)	** < 0.0001**
Body temperature (°C)	36.9 (0.3)	37.1 (0.3)	** < 0.001**
Blood glucose level (mg/dl)	90 (22)	69 (11)	** < 0.0001**
Bilirubin level (mg/dl)	7.2 (2.2)	9.3 (3.5)	**0.0014**
Venous hematocrit (%)*	48 (7)	55 (7)	** < 0.0001**
Lactate (mmol/l)*	1.9 (0.6)	2.2 (0.7)	0.1
Blood gases *			
pH	7.37 (0.05)	7.39 (0.03)	**0.02**
pCO_2_ (mmHg)	41 (7)	38 (5)	0.07
Base excess (mmol/l)	− 1.4 (2.3)	− 1.7 (1.6)	0.5
HCO_3_^−^ (mmol/l)	22.6 (1.8)	22.4 (1.5)	0.48

*values of venous samples only: preterm n = 29, term n = 43. Data are presented as mean (SD). Statistical analysis was performed with t-test or Mann–Whitney-U test. Statistically significant results are marked in bold.

**Table 3:** Microcirculatory parameters of premature and mature neonates obtained at 36-72 h of life.

	Premature neonates (n = 24)	Mature neonates (n = 85)	P value
PBR (µm)	1.9 (0.21)	2.14 (0.25)	**0.0001**
RBC-filling (%)	75.5 (7.7)	70.1 (7.4)	**0.0023**
TVD (µm/mm^2^)	1485 (353)	1286 (227)	**0.0017**
VVD (µm/mm^2^)	947 (289)	687 (146)	** < 0.0001**
Median RBCW (µm)	12.2 (2.2)	11.75 (1.3)	0.14

Data are presented as mean (SD). For VVD and TVD values of one preterm infant are missing due to technical problems during measurement. Statistical analysis was performed with t test. Statistically significant results are marked in bold.

### Microcirculatory measurements of preterm neonates

In 24 of the 39 premature infants, the first measurement was performed between 36 and 72 h of life as intended by protocol resulting in a mean PBR of 1.9 ± 0.21 µm. In the remaining 15 participants, the first measurement was postponed mostly due to clinical instability in the first postnatal days and delayed parental consent. PBR values were evenly distributed among infants (Suppl. Figure S3).

Table 2 depicts differences in clinical and laboratory data for preterm and term neonates at first measurement.

**Figure 3:**
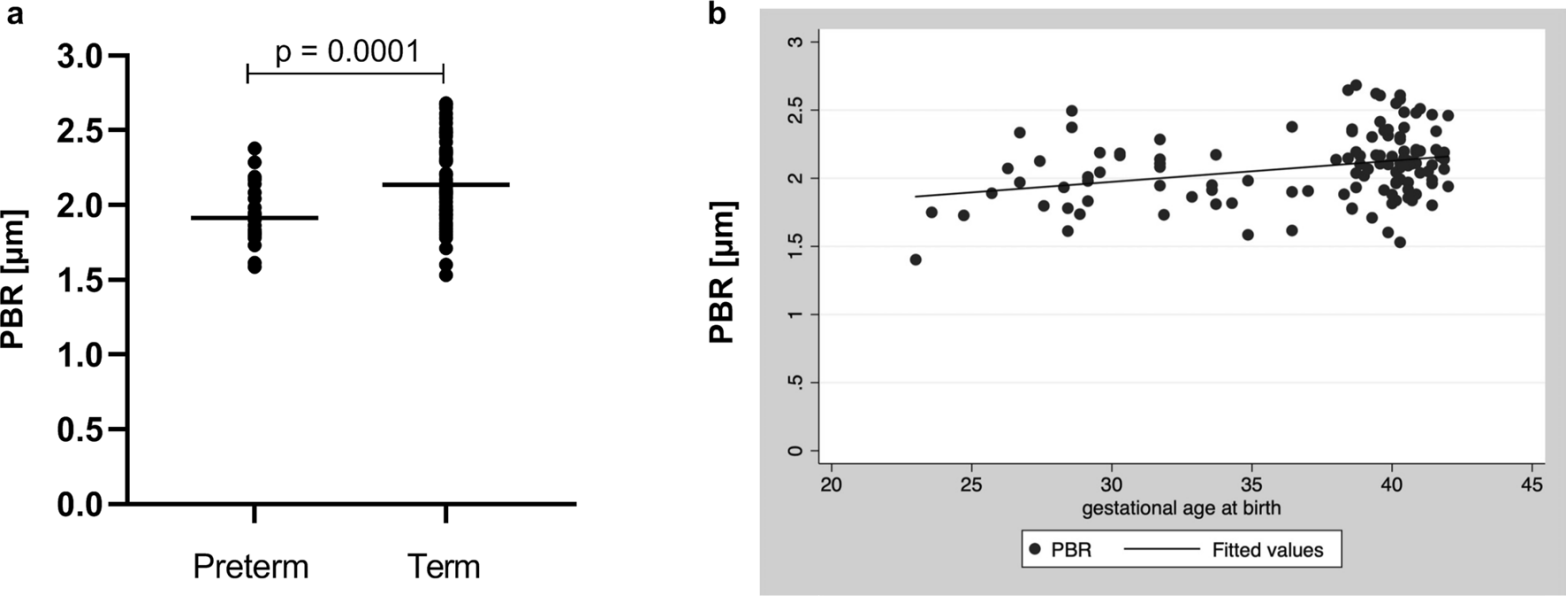
(**a**) PBR values obtained at 36–72 h of life in preterm (n = 24) and term (n = 85) neonates. (**b**) Linear regression showing association of PBR at first measurement and gestational age at birth (p < 0.001 adjusted for postnatal age).

Comparison of PBR values obtained at 36–72 h of life demonstrated significant differences between the study groups with significantly lower PBR values representing larger EG dimensions in the cohort of premature neonates (Table 3; Fig. 3a). Interestingly, there were also significant differences in RBC-filling, and vessel densities, with higher valid and total vessel density and larger RBC-filling in preterm neonates. As we were primarily interested in the association of PBR with gestational age at birth, we next used a sequential linear regression model to adjust for differences in postnatal age at first measurement and other potential confounders (i.e., parameters with a significant difference between the two groups as presented in Table 2). All covariates included had no effect on PBR except for postnatal age at measurement, which was therefore the only variable that was included in our final linear regression model (Suppl. Table S2). This analysis revealed a highly significant association between PBR and gestational age at birth adjusting for postnatal days of life, indicating that the EG thickness decreases with advancing gestational age (Fig. 3b). After stratification for term and preterm subgroups, the association of PBR with gestational and postnatal age remained significant only in Group A, suggesting that the effect of gestational age as well as postnatal age on PBR is more relevant in extremely premature neonates (Suppl. Table S3).

### Longitudinal evaluation of PBR in preterm neonates

**Figure 4:**
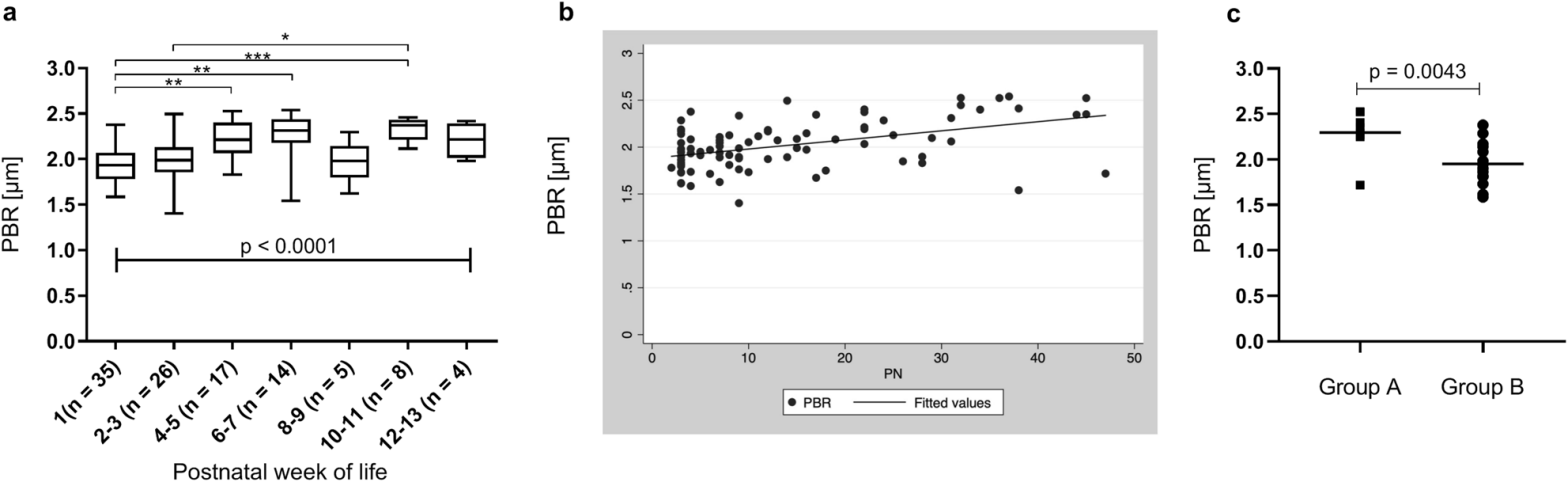
(**a**) PBR values obtained during longitudinal follow up of preterm neonates grouped according to postnatal age at measurement. (**b**) Linear mixed-effects model showing association of PBR and postnatal days (PN) per group. (**c**) Comparison of PBR values obtained in extremely premature neonates (Group A) when reaching a corrected gestational age > 30 + 0 weeks and PBR values in moderately preterm neonates (Group B) measured after birth. *p < 0.05, **p < 0.01, *p < 0.001.

To assess the effect of postnatal development on PBR, weekly follow up measurements were performed if possible. In total, 109 follow up measurements were obtained in 21 neonates, with an average number of 3.2 (range 1–9) measurements per child. In a first approach, all measurements were stratified according to their postnatal age, independent of the gestational age of the children. Due to low n-numbers for single measurement time points, especially beyond the third postnatal week, the results of two adjacent time points were combined. As shown in Fig. 4a, a significant increase in PBR was observed with advancing postnatal age. To account for the variation in gestational age, we next fitted a linear mixed-effects regression model including only the first four measurements per child to guarantee large enough group sizes. The model supports a statistically significant time-dependent variation with days of life, i.e., PBR is rising with postnatal age, adjusting for gestational age at birth (Fig. 4b; Suppl. Table S4).

To identify possible confounders of the association of postnatal age and PBR, we sequentially adjusted the linear mixed-effect model for clinical parameters with significant change over time (Suppl. Table S5). Here, hematocrit proved to be an independent predictor of PBR, i.e. PBR is increasing with decreasing hematocrit. After adjusting for capillary/venous hematocrit and gestational age at birth, we could still observe a linear increase of PBR with increasing postnatal age (Suppl. Table S4). All other variables (blood sugar, body temperature, MAP, bilirubin, pH and body weight) did not affect the association of PBR and time.

To further assess the impact of intrauterine versus extra uterine development on the EG, we compared the data of preterm group A when reaching a corrected gestational age of > 30 + 0 weeks with the values obtained in group B after birth. Thus, measurements in both groups were obtained at the same gestational age (group A: 33.2 ± 1.1 weeks vs. group B: 33.9 ± 1.7 weeks, p = 0.37), however at different postnatal ages (group A: 44 ± 4 days vs. group B: 3.3 ± 0.5 days, p < 0.001). Interestingly, we found that the extremely premature neonates had significantly higher PBR values at that time point than the moderately premature neonates after birth despite having the same corrected gestational age (Fig. 4c) and even higher PBR values than term neonates after birth (Group A: 2.3 ± 0.24 µm vs. term neonates 2.14 ± 0.25 µm, p = 0.028).

## Discussion

The EG is a complex, highly versatile structure that has essential roles in vascular integrity and function, and destruction of the EG has been described in the pathogenesis of micro- and macrovascular diseases^27^. Prematurity and low birth weight are known risk factors for cardiovascular disease, however, the exact mechanisms underlying this increased risk profile are still insufficiently understood, and the contribution of the EG remains elusive^1^. The aim of the present study was to describe the state of the EG in premature and mature neonates at birth and in the postnatal period, and to look at the effects of gestational age versus postnatal age on EG dimensions.

In the group of mature neonates representing a normal collective of our newborn ward, mean PBR measured between 36 and 72 h of age was 2.14 µm (median 2.11). Recently, values for PBR in young healthy adults were published, with a median PBR of 1.82 µm and thus presumably larger EG dimensions^28^. However, data from various studies evaluating PBR reveal large inter-individual variations in PBR. This was also true for the present study with PBR values in the relatively homogenous group of mature infants ranging from 1.53 to 2.68 µm. Thus, individual values need to be interpreted with caution, and larger cohorts need to be evaluated to define reference values for specific age groups.

In our cohort, we did not see an effect of birth mode or gender on PBR values, therefore these parameters were not considered in further analyses. As published before by Lee and colleagues, PBR values were significantly and inversely correlated with RBC-filling i.e., a larger PBR representing a smaller EG was associated with lower RBC-filling as a measure of microvascular perfusion^24^. The authors concluded that a thick “healthy” glycocalyx (small PBR) represented an efficient microvascular perfusion, whereas thinning of the glycocalyx (large PBR) was associated with impaired perfusion. As both parameters are assessed using the same algorithm to detect red blood cells based on their contrast against the surrounding tissue, we feel that further data is needed to support this conclusion, e.g., by evaluation of microvascular perfusion using other techniques such as NIRS or laser doppler flowmetry.

Surprisingly, we found a significant positive association of PBR and gestational age at birth i.e., premature neonates in the lowest gestational age group had the largest EG dimensions. Multivariate analysis adjusting for variations in postnatal age at measurement and potential confounders proved an independent effect of gestational age on PBR. At first glance, this finding seems unexpected, because one might assume that extreme prematurity is associated with an “immature” glycocalyx. However, taking a second look, it becomes evident that the EG likely serves important roles for vascular development in the embryo and fetus. Synthesis of heparan sulfate glycosaminoglycans (HSGAG), one of the major constituents of the EG, is upregulated during the differentiation of embryonic stem cells into endothelial cells. Knock down of the HSGAG-modifying enzyme NSDT1 resulted in a significantly reduced expression of endothelial markers in vitro and aberrant vessel formation in zebrafish embryos^29^. Likewise, Henderson-Toth and colleagues were able to demonstrate in quail embryos, that the EG is present as soon as blood flow starts and its disruption led to impaired vascular remodeling as well as altered gene expression^30^. These studies point at the importance of the EG, and HSGAGs in particular, during developmental vasculogenesis and angiogenesis. As reviewed by Iozzo and San Antonio, Heparan sulfate proteoglycans (HSPG) act in concert with proangiogenic factors, mainly VEGF, to control vascular development by providing a depot for these factors, limiting their diffusion and supporting receptor-ligand interaction^31^. More recently, Endomcuin-1 (EMCN), a sialoglycoprotein present in the EG of capillaries and veins, has been identified to regulate vascularization by modulating internalization of VEGF receptor 2^32,33^. Thus, a larger EG in lower gestational age premature neonates compared to moderate preterm and term infants might be expression of the ongoing vasculogenic activity at this developmental stage.

Furthermore, we explored the postnatal development of the PBR. We found that PBR in preterm neonates is rising with increasing postnatal age indicating that the glycocalyx thickness is reduced. Since gestational age and thus intrinsic maturational effects inevitably intermingle with extrinsic postnatal factors, we applied a linear mixed-effects regression analysis to separate these factors. The analysis revealed that postnatal age independently of advancing gestational age is a significant predictor of PBR. In addition, a postnatal drop in the hematocrit seems to account at least to some degree for the longitudinal changes in PBR. To further dissect intrinsic and extrinsic effects, we compared the follow-up values of a subgroup of extremely premature neonates (mean gestational age at birth 26.6 weeks) when reaching a corrected gestational age of > 30 completed weeks (postnatal day 36–47) with the postnatal values of moderately premature neonates born > 30 + 0 weeks of gestation (postnatal day 3–4). This analysis showed that PBR values in extremely premature infants obtained in postnatal week 6–7 were significantly higher than values in moderately premature infants after birth, despite having the same corrected gestational age. Values even exceeded those of mature neonates after birth. The magnitude of the observed PBR changes is comparable to that of other studies describing glycocalyx damage in critical illness such as sepsis and cardiac surgery with increases in PBR of 0.2–0.3 µm^18,20,34^. This is equivalent to about 1/3 of the total glycocalyx thickness measured in human and mouse capillaries^19,35,36^. This finding suggests that the normal intrinsically determined course of PBR development is accelerated and enhanced by extrauterine life. Given that the EG is a highly vulnerable structure that is shed in conditions such as hyperglycemia, sepsis and changes in volume status^34,37–39^, all of which are often present in ELBW neonates, our observation does not seem surprising.

The PBR assesses the inner part of the EG, which is made up of HSGAG to a large extent contributing substantially to EG thickness and structure^40^. Accordingly, EG loss as seen by an increase in PBR has been shown to go along with an increase in circulating HSPG^41^. HSPG are crucial for the mechanotransduction of shear stress regulating endothelial release of NO, and heparinase induced shedding leads to impaired flow mediated vasodilation^42^. Impaired endothelial function is a hallmark of vascular disease and has been demonstrated in young adult ex ELBW preterm neonates^15^ as well as in children with low birth weight^12^. Accelerated and enhanced postnatal reduction of the EG in ELBW neonates in our study could very well contribute to these long lasting alterations in endothelial function and thus increased vascular risk.

More than 20 years ago, it was already suggested that early impairment of vascular structure in fetal life might not be reversible and thus contribute to long term vascular sequelae^43^. In line with this notion, we have been able to show that preterm neonates have a characteristic vascular phenotype with increased vessel density that persists into adolescence^6,11^. In support of our previous results, the present study also showed higher vessel densities in preterm neonates compared to term infants. Regarding the differences in PBR between premature and mature neonates, our previous finding of a larger proportion of small vessels in premature neonates needs to be questioned. As the measurement of vessel diameters in SDF-recordings relies on the width of the red blood cell column, it might be that the impression of smaller vessel diameters is just a measurement artifact caused by differences in EG dimensions. If the EG is thicker, red blood cells are confined to the center of the vessel resulting in a smaller perfused diameter, despite the same anatomical vessel diameter. In the present study, the median diameter (P50) did not differ between premature and mature infants.

Our study has several limitations. First of all, the number of included premature neonates is small, and we were only able to obtain follow up measurements in half of the children due to various reasons such as clinical instability, transfer to another unit or discharge of the infant. As demonstrated by Gonzáles et al., measurements in the intensive care setting are limited by many factors such as instable respiratory state of the patients and lack of collaboration which is especially true for the neonatal and pediatric population^44^. The exclusion of sicker infants is likely to bias the results. However, as argued before, the EG is susceptible to many harmful stresses, and the inclusion of critically ill neonates might also introduce unmeasurable confounders. To not further reduce our numbers, we did not exclude infants treated for suspected infection, despite the known influence of sepsis on the EG. However, the diagnosis was solely based on elevated laboratory parameters, and none of the infants had culture-proven sepsis or was rated as being clinically septic.

Furthermore, differences in antenatal steroid exposure of premature neonates might have influenced our results, since hydrocortisone has been shown to protect the glycocalyx from degradation^45^. As we did not obtain corresponding data in the group of mature neonates, we were not able to include this possible confounder in the multivariate analysis. However, comparing the PBR values of neonates of group B exposed to antenatal steroids with those not exposed, did not yield significant differences, therefore is seems unlikely that this explains all of the age-dependent differences observed in the study.

Follow up in mature neonates to assess the natural postnatal course in this age group was not possible as these children are usually discharged within the first days of life, and ambulatory visits for study purposes are challenging to organize and not willingly accepted by the parents. We were able to obtain data from 85 mature and 39 premature neonates, demonstrating that SDF-based measurements of the glycocalyx are feasible in this cohort and can even be performed in ELBW neonates. The automated measurements using the GlycoCheck Software yielded acceptable interrater and intrarater variability. As discussed before, PBR measurements only assess the luminal part of the EG without giving information about its total thickness or composition. In addition, the measurements are limited to small and medium vessels of the cutaneous microcirculation, therefore only allowing assumptions on the global state of the EG. Due to the descriptive nature of the study, the mechanisms underlying the observed differences in PBR and the consequences for vascular function remain speculative. Still, the body of evidence is rising, that increases in PBR are paralleled by increases in circulating EG constituents indicating shedding of the glycocalyx and correlate with disease severity and adverse clinical outcome^34,41,46,47^. Thus non-invasive measurements of the glycocalyx remain a promising tool for easy and safe assessment of the EG in children.

In conclusion, we identified significant differences in the EG in premature and mature neonates. The natural course of EG development seems to underlie intrinsic regulation that is changed by extra-uterine factors in the setting of extreme prematurity. Thus, the study adds another piece to our understanding of the vascular peculiarities associated with premature birth. However, the puzzle is still far from being completed. Further studies are necessary to clarify to what extent and how changes in the EG possibly contribute to the increased cardiovascular risk in this patient group.

## Supplementary Information

Supplementary Information 1.

Supplementary Information 2.
