# Supplementary material for: Effect of gestational age and postnatal age on the endothelial glycocalyx in neonates
Source: Sci Rep. 2021 Feb 4;11:3133. doi: 10.1038/s41598-021-81847-8 (PMC7862677; doi:10.1038/s41598-021-81847-8)
Supplement: Supplementary file 1 — Supplementary Information 1. [file 41598_2021_81847_MOESM1_ESM.docx]

**Effect of gestational age and postnatal age on the endothelial glycocalyx in neonates**

Alexandra Puchwein-Schwepcke^1^, Stefanie Artmann^1^, Lea Rajwich^1^, Orsolya Genzel-Boroviczény^1^ and Claudia Nussbaum^1^

1 Division of Neonatology, Dr. von Hauner Children’s Hospital, University Hospital, LMU Munich

**Supplemental Tables**

| \|  \| Group A  (n = 20) \| Group B  (n = 19) \| P value \| \| --- \| --- \| --- \| --- \| \| Gestational age at birth (weeks) \| 27.5 (1.95) \| 33.2 (1.95) \| **<0.001** \| \| Postnatal day at first measurement \| 6.9 (5.4) \| 4.6 (2.8) \| 0.1 \| \| Birth weight (g) \| 995 (286) \| 1944 (484) \| **< 0.001** \| \| Female gender (n) \| 15 (75%) \| 8 (42%) \| 0.056 \| \| C-section (n) \| 17 (85%) \| 11 (58%) \| 0.08 \| \| Antenatal steroids (n) \| 20 (100%) \| 13 (68%) \| **0.008** \| \| Body temperature (°C) \| 36.9 (0.3) \| 37.0 (0.3) \| 0.19 \| \| Blood glucose level (mg/dl) \| 95 (22) \| 84 (19) \| 0.12 \| \| Bilirubin (mg/dl) \| 6.0 (1.4) \| 8.8 (1.7) \| **<0.001** \| \| Venous hematocrit (%) \| 48 (9) \| 49 (5) \| 0.82 \| \| pH \| 7.36 (0.05) \| 7.39 (0.04) \| **0.048** \|   Suppl. Table 1: Subgroups of preterm neonates  Group A: gestational age at birth < 30+0 weeks, Groups B: gestational age at birth ≥ 30+0 – 36+6 weeks.  Data are presented as mean (SD), or as number (%) as appropriate. Statistically significant results are marked in bold. |
| --- | --- | --- | --- | --- | --- | --- | --- | --- | --- | --- | --- | --- | --- | --- | --- | --- | --- | --- | --- | --- | --- | --- | --- | --- | --- | --- | --- | --- | --- | --- | --- | --- | --- | --- | --- | --- | --- | --- | --- | --- | --- | --- | --- | --- | --- | --- | --- | --- |

Suppl. Table 2: Sequential linear regression model of PBR values at first measurement testing the association between gestational age at birth and PBR

|  | | n of observations | ß-coefficient of association (95%CI) | p-value | ß-coefficient of covariate (95%CI) | p-value |
| --- | --- | --- | --- | --- | --- | --- |
| **Crude model*** | | 124 | 0.015 (0.007 - 0.024) | **0.001** | - | - |
| **Base model**** | | 124 | 0.023 (0.013 - 0.033) | **<0.001** | 0.026 (0.008 - 0.044) | **0.005** |
| **Base model adjusted for** | |  |  |  |  |  |
|  | blood sugar | 117 | 0.021 (0.009 - 0.033) | **0.001** | -0.001 (-0.004 - 0.002) | 0.484 |
|  | body temperature | 123 | 0.024 (0.013 - 0.034) | **<0.001** | -0.031 (-0.192 - 0.129) | 0.700 |
|  | bilirubin | 112 | 0.021 (0.010 - 0.032) | **<0.001** | 0.011 (-0.003 - 0.025) | 0.132 |
|  | venous hematocrit | 73 | 0.023 (0.010 - 0.037) | **0.001** | -0.001 (-0.010 - 0.008) | 0.844 |
|  | pH | 119 | 0.022 (0.012 - 0.033) | **0.001** | 0.391 (-0.718 – 1.501) | 0.486 |
|  | body weight | 124 | 0.023 (0.002 - 0.04) | **0.031** | 0.000 (-0.000 - 0.000) | 0.946 |
| *Crude model: testing the association between gestational age and PBR | | | | | | |
| **Base model: testing the association between gestational age and PBR, adjusting for postnatal days of life  For all models, the assumptions of normality, homoscedasticity, linearity and undue influence (cook’s distance) were tested and hold true. | | | | | | |

Suppl. Table 3: Linear regression of PBR values at first measurement – subgroup analysis adjusted for gestational age at birth and postnatal age

|  |  |  |  |  |  |
| --- | --- | --- | --- | --- | --- |
| **Group A < 30+0 weeks of gestation** | | |  |  |  |
| **Characteristic** | | **Crude association (95% CI)** | **p-value** | **adjusted association (95% CI)** | **p-value** |
| **n of observations** | | 20 |  | 20 |  |
| **GA at birth** | ß-coefficient | 0.061 ( -0.000 - 0.121) | 0.051 | 0.087 (0.036 - 0.137) | **0.002** |
| **postnatal days** | ß-coefficient | 0.020 ( -0.002 - 0.042) | 0.075 | 0.030 (0.012 - 0.048) | **0.003** |
|  |  |  |  |  |  |
| **Group B: ≥ 30+0 – 36+6 weeks of gestation** | | |  |  |  |
| **Characteristic** | | **Crude association (95% CI)** | **p-value** | **adjusted association (95% CI)** | **p-value** |
| **n of observations** | | 19 |  | 19 |  |
| **GA at birth** | ß-coefficient | -0.040 (-0.093 - 0.013) | 0.13 | -0.026 (-0.090 - 0.038) | 0.395 |
| **postnatal days** | ß-coefficient | 0.027 (-0.009 - 0.064) | 0.135 | 0.018 (-.027 - 0.062) | 0.411 |
|  |  |  |  |  |  |
| **term: ≥ 37+0 weeks of gestation** | | |  |  |  |
| **Characteristic** | | **Crude association (95% CI)** | **p-value** | **adjusted association (95% CI)** | **p-value** |
| **n of observations** | | 85 |  | 85 |  |
| **GA at birth** | ß-coefficient | 0.011 (-0.038 - 0.059) | 0.665 | 0.010 (-0.038 - 0.059) | 0.671 |
| **postnatal days** | ß-coefficient | 0.036 (-0.093 - 0.165) | 0.582 | 0.036 (-0.094 - 0.166) | 0.587 |

Suppl. Table 4: Linear mixed-effects regression model testing the association between postnatal days and PBR

|  | | n of observations | ß-coefficient of association (95%CI) | p-value | ß-coefficient of covariate (95%CI) | p-value |
| --- | --- | --- | --- | --- | --- | --- |
| **Crude model*** | | 85 | 0.008 (0.005 - 0.010) | **<0.001** | - | - |
| **Base model**** | | 85 | 0.008 (0.005 - 0.012) | **<0.001** | 0.012 (-0.008 - 0.031) | 0.235 |
| **Final model***** | | 85 | 0.005 (0.001 - 0.008) | **0.008** | GA: 0.027 (0.006 - 0.048) | **0.01** |
|  |  |  |  |  | Hct: -0.012 (-0.017 - -0.006) | **<0.001** |
| **Base model adjusted for** | |  |  |  |  |  |
|  | body weight | 85 | 0.011 (0.006 - 0.016) | **<0.001** | -0.000 (-0.000 - 0.000) | 0.121 |
|  | body temperature | 85 | 0.008 (0.005 - 0.012) | **<0.001** | -0.021 (-0.100 - 0.056) | 0.593 |
|  | mean arterial pressure | 80 | 0.009 (0.005 - 0.013) | **<0.001** | -0.001 (-0.006 - 0.004) | 0.776 |
|  | blood sugar | 82 | 0.009 (0.005 - 0.013) | **<0.001** | 0.001 (-0.001 - 0.003) | 0.239 |
|  | bilirubin | 83 | 0.008 (0.004 - 0.012) | **<0.001** | -0.005 (-0.031 - 0.021) | 0.708 |
|  | venous hematocrit | 63 | 0.003 (-0.001 - -0.007) | 0.091 | -0.013 (-0.020 - -0.007) | **<0.001** |
|  | capillary/venous hematocrit | 83 | 0.005 (0.001 - 0.008) | **0.008** | -0.012 (-0.017 - -0.006) | **<0.001** |
|  | lactate | 83 | 0.009 (0.006 - 0.013) | **<0.001** | 0.053 (-0.023 - 0.129) | 0.183 |
|  | pH | 83 | 0.009 (0.005 - 0.013) | **<0.001** | -0.404 (-1.279 - 0.472) | 0.366 |
| *Crude model: testing the association between postnatal days and PBR | | | | | | |
| **Base model: testing the association between postnatal days and PBR, adjusting for gestational age (GA) | | | | | | |
| ***Final model: testing the association between postnatal days and PBR, adjusting for GA and capillary/venous hematocrit (Hct) | | | | | | |

Suppl. Table 5) Postnatal changes of clinical and laboratory parameters in the group of premature neonates

|  | First measurement | Last measurement | p-value* |
| --- | --- | --- | --- |
| Postnatal day of life | 6.8 (5.3) | 26.3 (13) | **< 0.0001** |
| Body weight (g) | 1086 (114) | 1439 (454) | **< 0.0001** |
| Body temperature (°C) | 36.8 (0.3) | 36.8 (0.7) | 0.95 |
| Heart rate (bpm) | 157 (14) | 161 (14) | 0.17 |
| Respiratory rate (n/min) | 49 (13) | 49 (15) | 0.80 |
| Mean arterial pressure (mmHg) | 41 (7) | 47 (8) | **0.0115** |
| Oxygen Saturation (%) | 96 (3) | 96 (4) | 0.37 |
| Blood glucose level (mg/dl) | 96 (24) | 79 (13) | **0.0259** |
| Bilirubin level (mg/dl) | 6.0 (1.9) | 4.4 (2.8) | **0.0055** |
| Hematocrit (%) | 47 (8) | 41 (8) | **0.0093** |
| Lactate (mmol/l) | 1.9 (0.6) | 1.4 (0.4) | **0.0095** |

* all significantly differing covariates were included in the mixed-effects linear regression analysis.

Only premature neonates with ≥ 2 measurements were analyzed (n = 21).

For one infant, laboratory data is missing. Data are presented as Mean (SD).

Statistical analysis was performed using a Wilcoxon test.
