# Supplementary material for: Effect of gestational age and postnatal age on the endothelial glycocalyx in neonates
Source: Sci Rep. 2021 Feb 4;11:3133. doi: 10.1038/s41598-021-81847-8 (PMC7862677; doi:10.1038/s41598-021-81847-8)
Supplement: Supplementary file 2 — Supplementary Information 2. [file 41598_2021_81847_MOESM2_ESM.docx]

**Effect of gestational age and postnatal age on the endothelial glycocalyx in neonates**

Alexandra Puchwein-Schwepcke^1^, Stefanie Artmann^1^, Lea Rajwich^1^, Orsolya Genzel-Boroviczény^1^ and Claudia Nussbaum^1^

1 Division of Neonatology, Dr. von Hauner Children’s Hospital, University Hospital, LMU Munich

**Supplemental Figures**


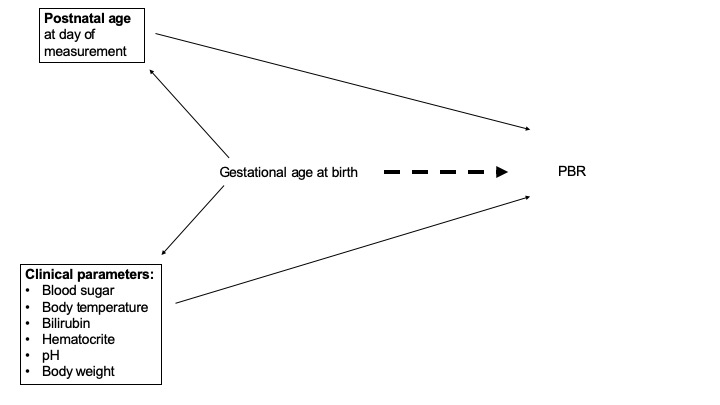


Suppl. Figure 1: The directed acyclic graph illustrates the possible association between gestational age at birth and PBR and its relationship to possible confounders that could interfere with this association. We decided to control for these predefined confounders by means of a sequential linear regression model and only included statistically significant confounders into our final multivariable linear regression model.


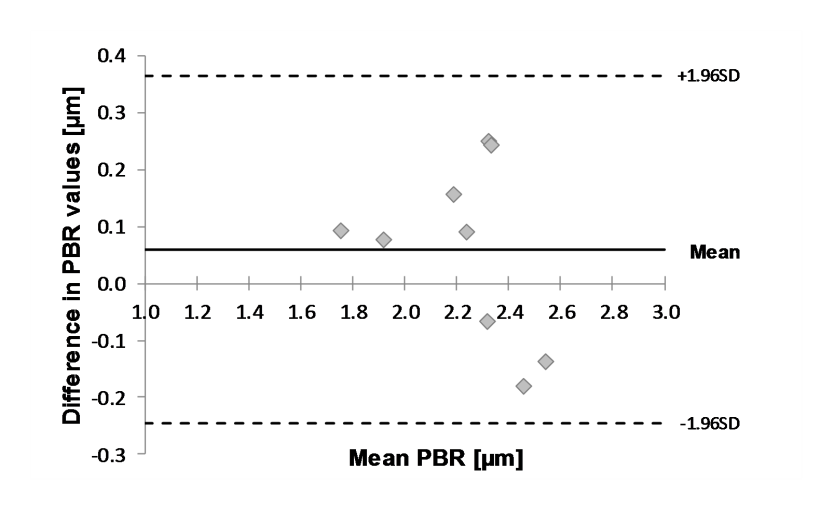


Suppl. Figure 2: Bland-Altman Diagram of PBR Values obtained by two different raters.


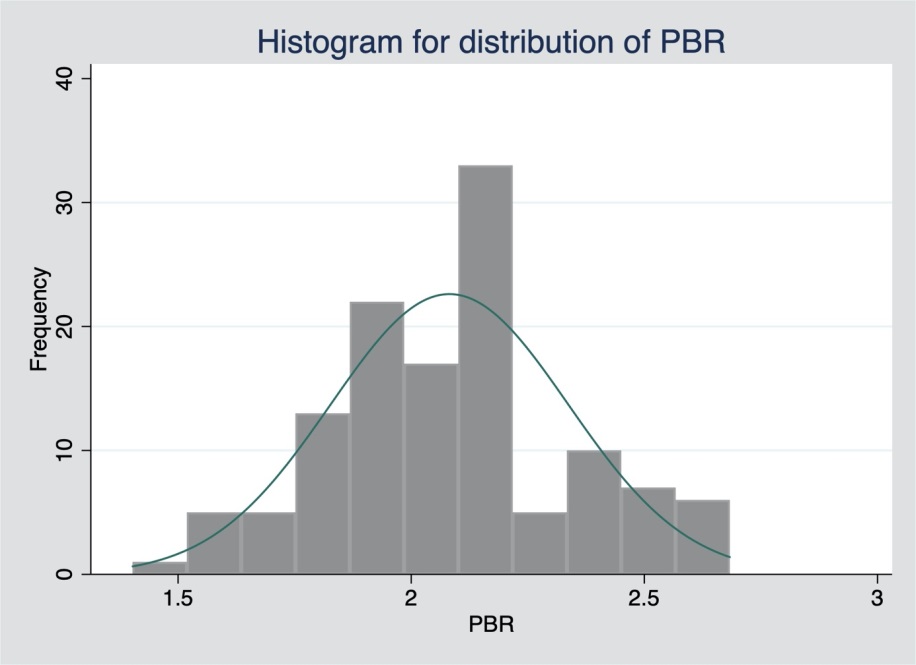


Suppl. Figure 3: Histogram of the distribution of PBR values at first measurement after birth in premature and mature neonates. In black you can see the Bell curve of a normal distribution.
